# Improved Retinal Microcirculation After Cardiac Surgery in Patients With Congenital Heart Disease

**DOI:** 10.3389/fcvm.2021.712308

**Published:** 2021-08-31

**Authors:** Cong Li, Zhuoting Zhu, Haiyun Yuan, Pingting Zhong, Qingsheng Peng, Xinran Dong, Manqing Huang, Baoyi Liu, Yun Ren, Yu Kuang, Xiaomin Zeng, Honghua Yu, Xiaohong Yang

**Affiliations:** ^1^Department of Ophthalmology, Guangdong Eye Institute, Guangdong Provincial People's Hospital, Guangdong Academy of Medical Sciences, Guangzhou, China; ^2^School of Medicine, South China University of Technology, Guangzhou, China; ^3^Guangdong Provincial Key Laboratory of South China Structural Heart Disease, Department of Cardiovascular Surgery, Guangdong Cardiovascular Institute, Guangdong Provincial People's Hospital, Guangdong Academy of Medical Sciences, Guangzhou, China; ^4^Medical College, Shantou University, Shantou, China; ^5^The Second School of Clinical Medicine, Southern Medical University, Guangzhou, China

**Keywords:** congenital heart disease, cardiac surgery, retinal vessel density, optical coherence tomography angiography, microcirculation

## Abstract

**Background:** Microcirculatory changes in congenital heart disease (CHD) patients undergoing cardiac surgery are not fully understood. We aimed to investigate the changes of retinal microcirculation in CHD patients after cardiac surgery by optical coherence tomography angiography (OCTA) and explore the association between retinal microcirculation and surgical outcome.

**Methods:** This prospective observational study consisted of 71 CHD patients aged ≥6 years undergoing cardiac surgery including 19 cyanotic CHD (CCHD) and 52 acyanotic CHD (ACHD). Optical coherence tomography angiography (OCTA) was used to measure vessel density (VD) and capillary density (CD) of radial peripapillary capillary (RPC) and peripapillary, VD of superficial capillary plexus (SCP) and deep capillary plexus (DCP), thickness of retinal nerve fiber layer (RNFL) and ganglion cell complex (GCC) preoperatively and 1 month postoperatively. Transthoracic echocardiography was conducted to measure macrocirculation.

**Results:** In CCHD patients, VD and CD of RPC and peripapillary increased postoperatively (all *P* < 0.05). In ACHD patients, VD of peripapillary, CD of RPC and peripapillary, and RNFL thickness increased postoperatively (all *P* < 0.05). VD of SCP and DCP, and GCC thickness did not change significantly in CHD patients after surgery. Lower preoperative retinal microvascular density was associated with longer cardiopulmonary bypass (CPB) time and postoperative length of stay (PLOS). No correlation was found between microcirculatory and macrohemodynamic parameters (all *P* > 0.05).

**Conclusions:** Improved retinal microcirculation was observed after congenital cardiac surgery and impaired preoperative retinal microvasculature was associated with prolonged CPB time and PLOS, which might provide potential information about the outcome of congenital cardiac surgery.

## Introduction

Although advances in the surgical treatment of congenital heart disease (CHD) have substantially improved the overall survival of patients, the perioperative management and prediction of the surgical outcome remain challenging (1, 2). It has been documented that microcirculation dysfunction was independently associated with adverse outcome in patients with valvular or coronary cardiac surgery using cardiopulmonary bypass (CPB), implying microcirculatory monitoring could provide the predictive value of surgical outcome (3, 4). Common techniques have been used to explore the changes of microcirculation including sublingual microscopy (5), laser Doppler perfusion monitoring (6), and near-infrared reflectance spectroscopy (7). Researches exploring new methods for accurately evaluating microcirculation in patients undergoing congenital cardiac surgery are of continual interest and need.

The retina and other end organs (such as the brain and kidney) share similar anatomical features and physiological properties, which allows direct non-invasive visualization of the body's microvasculature, offering a unique and easily accessible window to systemic microvascular pathology in patients undergoing cardiac surgery (8, 9). Optical coherence tomography angiography (OCTA) is a non-invasive, rapid imaging technique used to obtain high-resolution, three-dimensional images of the retinal microvasculature, and can be used to automatically quantify retinal microcirculatory perfusion at different layers (10). Given its excellent repeatability and reproducibility, OCTA has been widely used in clinical practice for monitoring microvascular function in hypertension (11), diabetes (12), and so on (13). In our previous study, a significant impairment of retinal microvasculature was found in the OCTA imaging of CHD patients (14). As for the alteration of postoperative retinal microvasculature, although it has been investigated in patients undergoing coronary artery bypass or valve replacement with CPB (15–18), there is little evidence regarding patients undergoing congenital cardiac surgery.

In this prospective observational study, we aimed to reveal the changes of postoperative retinal microcirculation in CHD patients and explore the association between preoperative retinal microcirculation and adverse outcome of congenital cardiac surgery.

## Materials and Methods

### Study Design and Patient Population

In this study, all CHD patients (aged ≥ 6 years) were scheduled for cardiac surgery with CPB between May 2017 and October 2020 in Guangdong Provincial People's Hospital. CHD patients were classified into acyanotic (ACHD, SaO_2_ > 90%) and cyanotic (CCHD, SaO_2_ ≤ 90%) based on the value of their preoperative resting oxygen saturation (SaO_2_) (19). All patients went through comprehensive ophthalmologic and cardiac examination prior to the surgery and 1 month after surgery. The examination included refraction measurement, best-corrected visual acuity (BCVA) test, intraocular pressure (IOP) measurement using a non-contact tonometer, OCTA, and transthoracic echocardiography.

The exclusion criteria were as follows: (1) did not perform CPB during surgery; (2) a history of glaucoma, uveitis, or retinal diseases; (3) IOP > 21 mmHg; (4) a history of intraocular surgery; (5) media opacities preventing high-quality imaging; (6) patients with preoperative comorbidities such as hypertension, diabetes, renal disease, and severe cardiovascular diseases.

All study procedures were conducted in accordance with the principles of the Declaration of Helsinki, and ethics approval was obtained from the Research Ethics Committee of Guangdong Provincial People's Hospital [No.GDREC2018148H(R1)]. The study protocol was explained in detail to all participants and written informed consent was obtained from participants (or their parent or legal guardian in the case of children under 16 years) prior to the study.

### Optical Coherence Tomography Angiography

Patients underwent an OCTA examination using an AngioVue OCTA system (Version 2017.1.0.151; RTVue-XR Avanti; Optovue, Fremont, CA, USA) which included a 6 × 6 mm^2^ high-definition (HD) macular scan and a 4.5 × 4.5 mm^2^ HD optic disc scan. The system uses a light source with peak wavelength at 840 nm and a bandwidth of 50 nm with a scanning speed at 70 kHz and can provide an axial tissue resolution of 5 μm.

The software of the RTVue-XR Avanti utilizes a projection-resolved OCTA algorithm to automatically segment the superficial capillary plexus (SCP), the deep capillary plexus (DCP), and the radial peripapillary capillary (RPC). A split-spectrum amplitude-decorrelation angiography (SSADA) software algorithm was used to automatically calculate retinal vessel density (RVD) defined as the percentage of the measured area occupied by flowing blood vessels (20). An orthogonal registration algorithm for correction of motion artifacts was employed to merge three-dimensional OCT angiograms.

In the optic disc, RPC is defined as the layer between the outer limit of the retinal nerve fiber layer (RNFL) and the internal limiting membrane (ILM). The software automatically fits a 2 mm diameter circle centered around the optic disc; the peripapillary region is defined as the area extending between the 2- and 4-mm-diameter elliptical contour lines around the optic disc boundary. Capillary density (CD) was measured using the built-in software that automatically removes larger vessels of diameter ≥ 3 pixels (~≥33 μm) during the 4.5 × 4.5 mm^2^ HD optic disc scan (11). The following parameters were measured at the optic disc: the VD and CD of RPC and peripapillary, and RNFL thickness.

In the macular region, SCP is defined as the ILM to 10 μm above the inner plexiform layer (IPL) and DCP is defined as 10 μm above the IPL to 10 μm below the outer plexiform layer (OPL). The ganglion cell complex (GCC) layer is defined as the layer extending from RNFL to IPL. The following parameters were measured at the macula: the VD of SCP and DCP, and GCC thickness.

For each participant, one eye was selected randomly, and the same eye was measured preoperatively and postoperatively. Poor quality images were excluded from the analysis. More specifically, an image was excluded if one of the following criteria were met: (1) quality index (QI) <6; (2) residual motion artifacts; (3) segmentation error; and (4) local weak signal caused by artifacts such as floaters.

### Surgical Procedures

Before the initiation of CPB, anticoagulation was achieved using heparin to maintain an activated clotting time ≥ 480 s. During CPB, diluted hematocrit was generally maintained at 25–30%. CPB was generally conducted under mild hypothermia (30–34°C) according to the surgical requirements. Standard ultrafiltration was performed routinely. Myocardial protection was achieved by intermittent antegrade modified St. Thomas cardioplegia. All surgical procedures were done by the same team, and then CHD patients were rewarmed to a bladder temperature >35°C before separation from CPB. After separation, heparin was neutralized by the administration of protamine. Patients were transferred to the intensive care unit (ICU) after surgery.

### Data Collection

Clinical, demographic, procedural, and outcome data were extracted and recorded from electronic patient files and ICU charts. Patient preoperative characteristics including age, sex, body mass index, blood pressure, hemoglobin, hematocrit, SaO_2_, and anatomic diagnosis were recorded. Intraoperative and postoperative data were collected including operation time, CPB time, aortic cross-clamp time, details on the procedure performed, mechanical ventilation time, length of ICU stay, and postoperative length of stay (PLOS). All participants underwent transthoracic echocardiography performed by trained sonographers before and 1 month after surgery, and measurements and analyses were performed by expert technicians and cross-checked by certified echocardiographers. Echocardiographic parameters collected included left ventricular ejection fraction (LVEF), left ventricular end-diastolic dimension (LVEDd), left ventricular end-systolic dimension (LVEDs), early mitral annular velocity (e'), and early transmitral inflow to early mitral relaxation velocity ratio (E/e').

### Statistical Analysis

Continuous variables are presented as mean and standard deviation (SD) or median and interquartile range (IQR) according to data distribution. Categorical variables are presented as a number (percentage). The normality of the data was tested using the Shapiro-Wilk test. Paired *t*-test was used to examine the change of postoperative RVD in the ACHD and CCHD patients. The change of RVD was compared between ACHD and CCHD patients using the student *t*-test. The 50th percentile of CPB time and PLOS were used to establish the cutoff value for defining prolonged CPB time and PLOS. Prolonged CPB time was used as an essential perioperative risk factor which has been confirmed to be associated with increased mortality and morbidity, and prolonged PLOS was used as an indicator for the surgical outcome. Student *t*-test was performed to compare the differences of RVD in patients with and without prolonged CPB time and PLOS. Receiver operating characteristic (ROC) analysis was carried out to determine the performance of OCTA parameters to identify patients with prolonged CPB time and PLOS. Spearman's correlation analysis was used to investigate the relationship between RVD and echocardiographic parameters. Statistical analysis was performed with SPSS (IBM SPSS, Version 25.0, IBM Corporation, Armonk, NY, USA). All *P*-values were from 2-sided tests and results were deemed statistically significant at *P* < 0.05.

## Results

### Patient Characteristics

A total of 82 CHD patients were consecutively enrolled in our study. Eight patients were excluded because CPB was not used and three patients were excluded because of poor OCTA image quality in the follow-up examination. Finally, a total of 71 patients with CHD (age range, 6–53 years; 41% male) were enrolled including 52 ACHD patients and 19 CCHD patients. The baseline demographic and clinical characteristics are summarized in Table 1. There was no significant change in BCVA and IOP after surgery (BCVA: 0.02 ± 0.05 logMAR vs. 0.02 ± 0.07 logMAR, *P* = 0.306; IOP: 14.83 ± 2.56 mmHg vs. 14.47 ± 3.10 mmHg, *P* = 0.369). The distribution of congenital cardiac lesions is shown in Table 2.

**Table 1:** Baseline characteristics of patients.

	**Total**	**ACHD**	**CCHD**
	**(*n* = 71)**	**(*n* = 52)**	**(*n* = 19)**
Age (years)	23 ± 10	24 ± 10	20 ± 9
Male, *n* (%)	29 (40.8)	20 (38.5)	9 (47.4)
Body mass index (kg/m^2^)	18.64 ± 3.51	19.15 ± 3.50	17.26 ± 3.20
Systolic BP (mmHg)	112 ± 12	114 ± 13	109 ± 9
Diastolic BP (mmHg)	70 ± 10	72 ± 10	65 ± 10
SE (diopters)	−2.15 ± 2.71	−1.92 ± 2.42	−2.82 ± 3.43
BCVA, LogMAR	0.02 ± 0.07	0.02 ± 0.07	0.03 ± 0.06
IOP (mmHg)	14.55 ± 3.04	14.64 ± 2.92	14.26 ± 3.47
SaO_2_ (%)	98 (89–100)	99 (97–100)	85 (78–87)
Hb (g/L)	145 (130–175)	137 (128–153)	180 (164–185)
HCT (L/L)	0.43 (0.39–0.51)	0.41 (0.38–0.45)	0.53 (0.49–0.61)
Previous sternotomy,*n* (%)	12 (16.9)	5 (9.6)	7 (36.8)
Operation time (minutes)	258 (198–306)	242 (180–280)	305 (270–370)
CPB time (minutes)	134 (103–174)	127 (89–163)	144 (119–205)
ACC time (minutes)	83 (54–110)	73 (55–98)	90 (54–141)
ICU stay (days)	2 (1–4)	2 (1–3)	4 (2–6)
MV time (hours)	13 (6–21)	10 (6–20)	18 (7–27)
Postoperative length of stay (days)	7 (6–12)	7 (5–10)	12 (8–15)

*Quantitative variables are expressed as mean ± standard deviation or median (interquartile range), and categorical variables are expressed as percentages of total*.

*ACHD, acyanotic congenital heart disease; ACC, aortic cross-clamp; BCVA, best-corrected visual acuity; BP, blood pressure; CCHD, cyanotic congenital heart disease; CPB, cardiopulmonary bypass; Hb, hemoglobin; HCT, hematocrit; ICU, intensive care unit; IOP, intraocular pressure; LogMAR, logarithm of the minimum angle of resolution; MV, mechanical ventilation; SaO2, oxygen saturation; SE, spherical equivalent*.

**Table 2:** Distribution of main cardiac malformations of CHD patients.

	**ACHD (*n* = 52)**	**CCHD (*n* = 19)**
Tetralogy of Fallot	6 (11.5)	6 (31.5)
Ebstein anomaly	16 (30.8)	1 (5.3)
Pulmonary atresia	0	3 (15.8)
Transposition of the great arteries	0	3 (15.8)
Heart septal defects	13 (20.0)	2 (10.5)
Double-outlet right ventricle	0	1 (5.3)
Single ventricle	0	1 (5.3)
Anomalous pulmonary venous connection	4 (7.7)	0
Right ventricular outflow tract stenosis	3 (5.8)	0
Other	10 (19.2)	2 (10.5)

*Values are expressed as percentages of total*.

*ACHD, acyanotic congenital heart disease; CCHD, cyanotic congenital heart disease; CHD, congenital heart disease*.

### Comparison of Preoperative and Postoperative RVD

**Figure 1:**
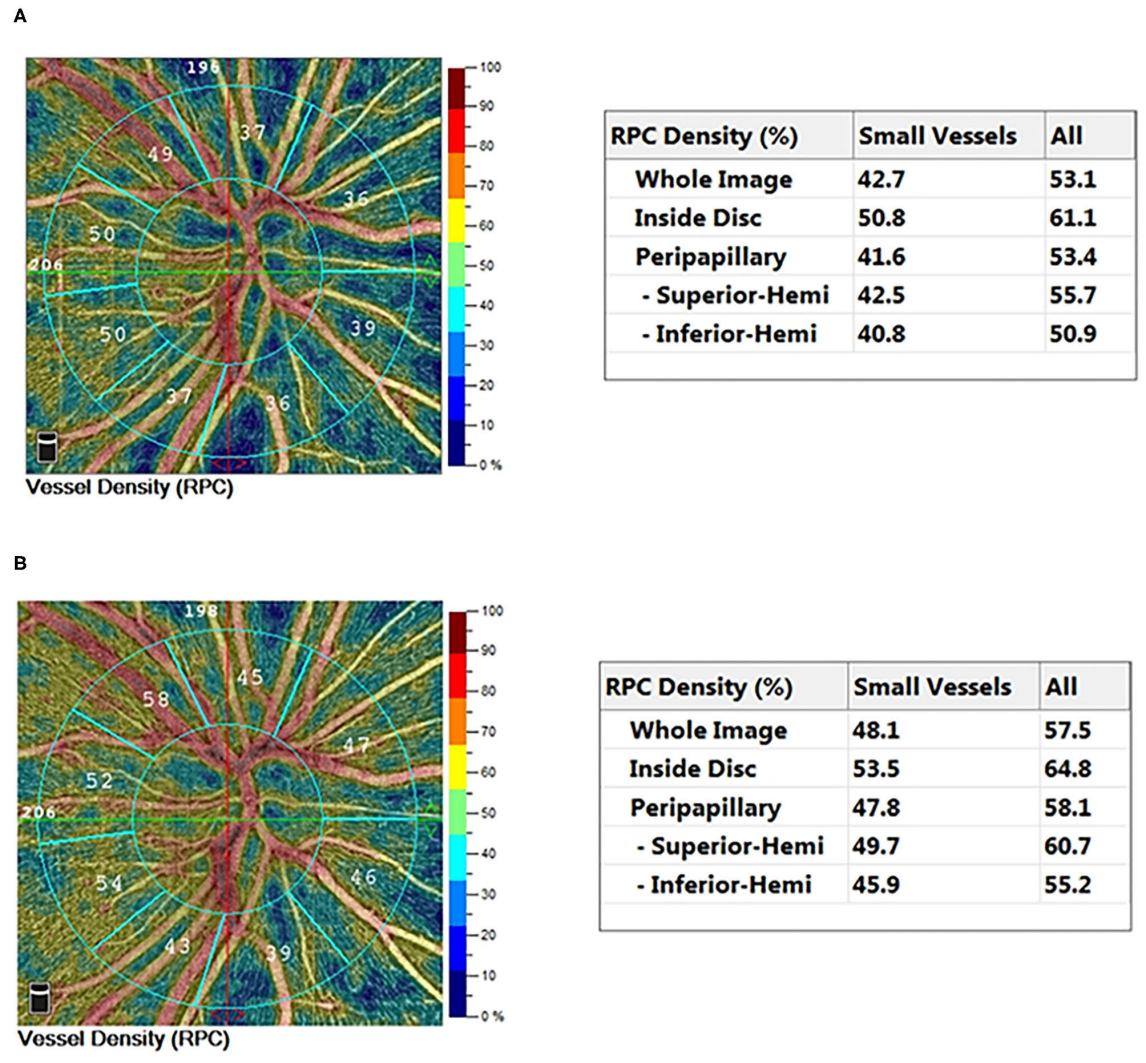
Typical 4.5 × 4.5 mm^2^ HD OCTA color-coded vessel density map of a cyanotic congenital heart disease eye preoperatively **(A)** and postoperatively **(B)**.

The VD and CD of RPC and peripapillary, and RNFL thickness were significantly increased in CHD patients after cardiac surgery (all *P* < 0.05). However, there were no significant differences in VD of SCP and DCP, and GCC thickness (all *P* > 0.05) (Table 3). In CCHD patients, a significant increase was observed in postoperative VD and CD of RPC and peripapillary (all *P* < 0.05) (Figure 1). However, no significant differences were found in VD of SCP and DCP, and thickness of RNFL and GCC (all *P* > 0.05). In ACHD patients, VD of peripapillary, CD of RPC and peripapillary, and RNFL thickness increased significantly after surgery (all *P* < 0.05). No significant differences were observed in VD of RPC, SCP and DCP, and GCC thickness (all *P* > 0.05) (Table 4). The changes in VD of RPC, and CD of RPC and peripapillary were found to be more significant in CCHD patients when compared with ACHD patients (all *P* < 0.05) (Table 5). Besides, the comparison of preoperative and postoperative RVD was also conducted in different age groups divided by median age (Table 6). The postoperative CD of RPC and peripapillary were significantly increased in younger patients (all *P* < 0.05), and the postoperative VD and CD of RPC and peripapillary were also significantly increased in older patients (all *P* < 0.05).

**Table 3:** Comparison of RVD before and after cardiopulmonary bypass in CHD patients.

	**Preoperative**	**Postoperative**	***P*-value**
RPC density (%)
Mean	55.23 ± 3.18	55.85 ± 2.96	0.019^*^
Peripapillary	56.42 ± 3.95	57.36 ± 3.53	0.002^*^
RPC capillary density (%)
Mean	47.57 ± 3.98	48.74 ± 3.27	<0.001^*^
Peripapillary	48.49 ± 5.06	50.20 ± 3.95	<0.001^*^
Macular vessel density (%)
Mean SCP	50.16 ± 2.86	50.33 ± 3.07	0.602
Mean DCP	51.26 ± 6.12	50.15 ± 6.66	0.180
GCC thickness (μm)	98.47 ± 7.57	99.18 ± 10.18	0.404
RNFL thickness (μm)	106.53 ± 9.34	107.55 ± 9.89	0.032^*^

*Values are presented as mean ± SD*.

*CHD, congenital heart disease; DCP, deep capillary plexus; GCC, ganglion cell complex; RNFL, retinal nerve fiber layer; RPC, radial peripapillary capillary; RVD, retinal vessel density; SCP, superficial capillary plexus*.

*Paired t-test was used to compare continuous variables*.

* *Significant statistical difference, P <0.05*.

**Table 4:** Comparison of retinal vessel density before and after surgery in CCHD and ACHD patients.

	**CCHD patients (** ***n*** **=** **19)**			**ACHD patients (** ***n*** **=** **52)**		
	**Preoperative**	**Postoperative**	***P*-value**	**Preoperative**	**Postoperative**	***P*-value**
RPC density (%)
Mean	52.89 ± 3.93	54.39 ± 4.46	0.015^*^	56.07 ± 2.40	56.43 ± 1.98	0.208
Peripapillary	53.23 ± 4.97	55.16 ± 5.19	0.017^*^	57.58 ± 2.78	58.22 ± 2.25	0.025^*^
RPC capillary density (%)
Mean	43.90 ± 4.84	46.70 ± 5.72	<0.001^*^	48.87 ± 2.57	49.51 ± 1.97	0.031^*^
Peripapillary	43.52 ± 6.08	47.28 ± 5.72	<0.001^*^	50.27 ± 3.11	51.31 ± 2.34	0.001^*^
Macular vessel density (%)
Mean SCP	49.28 ± 3.69	49.54 ± 3.84	0.720	50.49 ± 2.45	50.62 ± 2.72	0.708
Mean DCP	48.76 ± 6.68	48.47 ± 7.24	0.887	52.18 ± 5.69	50.77 ± 6.39	0.104
GCC thickness (μm)	96.43 ± 9.79	98.69 ± 17.26	0.489	99.19 ± 6.58	99.35 ± 6.29	0.561
RNFL thickness (μm)	105.46 ± 12.71	105.52 ± 13.79	0.956	106.88 ± 8.04	108.23 ± 8.29	0.011^*^

*Values are presented as mean ± SD*.

*ACHD, acyanotic congenital heart disease; CCHD, cyanotic congenital heart; DCP, deep capillary plexus; GCC, ganglion cell complex RNFL, retinal nerve fiber layer; RPC, radial peripapillary capillary; SCP, superficial capillary plexus*.

*Paired t-test was used to compare continuous variables*.

* *Significant statistical difference, P <0.05*.

**Table 5:** Comparison of retinal vessel density before and after surgery in CHD patients of different age groups (*n* = 71).

	**Age** **≤** **median (** ***n*** **=** **38)**			**Age** **>** **median (** ***n*** **=** **33)**		
	**Preoperative**	**Postoperative**	***P*-value**	**Preoperative**	**Postoperative**	***P*-value**
RPC density (%)
Mean	55.19 ± 3.07	55.61 ± 2.77	0.272	55.29 ± 3.35	56.13 ± 3.19	0.020^*^
Peripapillary	56.38 ± 3.66	57.03 ± 3.26	0.116	56.45 ± 4.32	57.73 ± 3.85	0.004^*^
RPC capillary density (%)
Mean	47.54 ± 4.05	48.58 ± 3.20	0.015^*^	47.60 ± 3.95	48.93 ± 3.38	0.004^*^
Peripapillary	48.43 ± 5.02	49.98 ± 3.79	0.002^*^	48.56 ± 5.19	50.45 ± 4.16	0.001^*^
Macular vessel density (%)
Mean SCP	49.45 ± 2.35	49.62 ± 3.16	0.637	50.99 ± 3.19	51.15 ± 2.78	0.775
Mean DCP	50.43 ± 5.77	48.50 ± 7.17	0.117	52.22 ± 6.46	52.06 ± 5.52	0.880
GCC thickness (μm)	98.33 ± 6.21	98.05 ± 6.50	0.385	98.62 ± 8.92	100.41 ± 13.08	0.310
RNFL thickness (μm)	107.25 ± 8.45	108.07 ± 9.64	0.205	105.65 ± 10.39	106.93 ± 10.32	0.081

*Values are presented as mean ± SD*.

*CHD, congenital heart disease; DCP, deep capillary plexus; GCC, ganglion cell complex RNFL, retinal nerve fiber layer; RPC, radial peripapillary capillary; SCP, superficial capillary plexus*.

*Paired t-test was used to compare continuous variables*.

* *Significant statistical difference, P <0.05*.

**Table 6:** Comparison of changes in retinal vessel density between CCHD and ACHD patients.

	**CCHD patients (*n* = 19)**	**ACHD patients (*n* = 52)**	***P*-value**
RPC density (%)
Mean	1.50 ± 2.42	0.30 ± 2.00	0.037^*^
Peripapillary	1.93 ± 3.21	0.58 ± 2.02	0.101
RPC capillary density (%)
Mean	2.81 ± 2.66	0.57 ± 2.11	<0.001^*^
Peripapillary	3.75 ± 3.71	0.96 ± 2.22	0.005^*^

*Values are presented as mean ± SD*.

*ACHD, acyanotic congenital heart disease; CCHD, cyanotic congenital heart; RPC, radial peripapillary capillary*.

*Student t-test was used to compare continuous variables*.

* *Significant statistical difference, P <0.05*.

### Comparison of RVD Between Patients With and Without Prolonged CPB Time and PLOS

Compared with patients without prolonged CPB time, the CD of RPC and peripapillary were significantly lower in patients with prolonged CPB time (both *P* < 0.05). No significant differences were found in VD of RPC and peripapillary (both *P* > 0.05). In patients with prolonged PLOS, VD of peripapillary and CD of RPC and peripapillary were significantly lower compared with patients without prolonged PLOS (all *P* < 0.05). No significant difference was found in VD of RPC (*P* > 0.05) (Table 7). The ROC showed the ability of RVD to distinguish patients with prolonged CPB time and PLOS (Supplementary Figure 1). The area under the receiver operating characteristic curve (AUC) of the VD of mean RPC, RPC capillary, peripapillary RPC and peripapillary RPC capillary to identify patients with prolonged CPB time is 0.679, 0.695, 0.670, and 0.702, respectively. The AUC of the VD of mean RPC, RPC capillary, peripapillary RPC and peripapillary RPC capillary to identify patients with prolonged PLOS is 0.611, 0.650, 0.626, and 0.676, respectively.

**Table 7:** Comparison of preoperative retinal vessel density in patients with and without prolonged CPB time and PLOS.

	**Prolonged CPB time**			**Prolonged PLOS**		
	**Yes (*n* = 34)**	**No (*n* = 37)**	***P*-value**	**Yes (*n* = 34)**	**No (*n* = 37)**	***P*-value**
RPC density (%)
Mean	54.50 ± 2.86	55.90 ± 3.36	0.064	54.49 ± 3.97	55.92 ± 2.06	0.067
Peripapillary	55.50 ± 3.34	57.26 ± 4.32	0.060	55.33 ± 4.99	57.41 ± 2.33	0.032^*^
RPC capillary density (%)
Mean	46.51 ± 3.70	48.55 ± 4.02	0.030^*^	46.44 ± 4.75	48.61 ± 2.79	0.025^*^
Peripapillary	47.18 ± 4.51	49.70 ± 5.30	0.035^*^	46.89 ± 6.04	49.96 ± 3.43	0.012^*^

*Values are presented as mean ± SD*.

*CPB, cardiopulmonary bypass; PLOS, postoperative length of stay; RPC, radial peripapillary capillary*.

*Student t-test was used to compare continuous variables*.

* *Significant statistical difference, P <0.05*.

### Correlation Between Microcirculatory and Echocardiographic Parameters

Regarding the relationship between microcirculatory and macrohemodynamic characteristics, no significant correlation was observed between RVD and LVEF, LVEDs, LVEDd, e', and E/e' ratio preoperatively and postoperatively (all *P* > 0.05) (Table 8).

**Table 8:** Correlation between microcirculatory and macrohemodynamic parameters in CHD patients.

**Microcirculatory parameters**	**Macrohemodynamic parameters**	**Preoperative**	**Postoperative**
RPC density, %	LV ejection fraction, %	(0.170, 0.162)	(0.089, 0.469)
	LV end-systolic diameter, cm	(−0.104, 0.403)	(−0.040, 0.747)
	LV end-diastolic diameter, cm	(−0.130, 0.293)	(−0.101, 0.417)
	e', cm/s	(0.185, 0.149)	(−0.035, 0.786)
	E/e'	(−0.157, 0.315)	(−0.163, 0.273)
RPC capillary density, %	LV ejection fraction, %	(0.123, 0.312)	(0.112, 0.363)
	LV end-systolic diameter, cm	(−0.055, 0.659)	(−0.035, 0.778)
	LV end-diastolic diameter, cm	(−0.100, 0.422)	(−0.115, 0.355)
	e', cm/s	(0.261, 0.051)	(0.036, 0.779)
	E/e'	(−0.193, 0.216)	(−0.256, 0.082)
Peripapillary density, %	LV ejection fraction, %	(0.207, 0.088)	(0.181, 0.139)
	LV end-systolic diameter, cm	(−0.102, 0.409)	(−0.114, 0.360)
	LV end-diastolic diameter, cm	(−0.100, 0.421)	(−0.156, 0.208)
	e', cm/s	(0.117, 0.367)	(−0.087, 0.498)
	E/e'	(−0.088, 0.576)	(−0.222, 0.133)
Peripapillary capillary density, %	LV ejection fraction, %	(0.145, 0.234)	(0.205, 0.094)
	LV end-systolic diameter, cm	(−0.062, 0.616)	(−0.080, 0.521)
	LV end-diastolic diameter, cm	(−0.084, 0.501)	(−0.122, 0.325)
	e', cm/s	(0.170, 0.188)	(−0.082, 0.525)
	E/e'	(−0.098, 0.533)	(−0.253, 0.087)

*Spearman's correlation coefficients and the P-values (in brackets)*.

*CHD, congenital heart disease; e', early mitral annular velocity; E/e', early transmitral inflow to early mitral relaxation velocity ratio; LV, left ventricular; RPC, radial peripapillary capillary*.

## Discussion

Results of our prospective study showed that RVD was improved after congenital cardiac surgery with CPB and revealed the association between preoperative impairment in RVD and prolonged CPB time and PLOS, which indicated the potential of retinal microcirculation in the assessment of outcome for patients with congenital cardiac surgery. Besides, no correlation was found between microcirculatory variables and macrohemodynamic parameters in CHD patients undergoing cardiac surgery.

Retinal perfusion was significantly increased in CCHD patients after cardiac surgery with CPB in our study. Similarly, previous studies have found that the retinal vascular morphology, such as dilatation and tortuosity, would return to normal after cardiac surgery in CCHD patients (21, 22). On the one hand, postoperative higher oxy-hemoglobin concentrations could enhance oxygen delivery to microcirculation (23, 24). On the other hand, reduced blood viscosity could change the shear stress and increase nitric oxide (NO) production and result in vascular smooth muscle cell relaxation and vasodilation after surgery (25, 26). A previous study examined the microcirculatory changes in CHD patients undergoing cardiac surgery with CPB and reported an increased sublingual microcirculatory perfusion in CCHD patients during ICU stay (27). However, preoperative and late postoperative microcirculatory parameters could not be studied due to the difficulty to use sublingual microscopy in extubated and awake patients.

In the present study, a large-vessel masking approach was selected to separate larger vessels from capillaries. The results showed that the increase of RVD was more notable in capillaries. This might result from variations in blood viscosity that could exert a more obvious effect at a capillary level compared to large vessels (28). In addition, large vessels make up a larger proportion of VD and have a substantial contribution to the total peripapillary perfusion, which might mask a change in VD (29). Several studies have also shown that subtraction of the peripapillary large vessels was associated with enhanced diagnostic accuracy of peripapillary perfusion for glaucoma (30) and diabetic retinopathy (31).

Our results also revealed that preoperative impairment in RVD was associated with longer CPB time and PLOS. Furthermore, the ROC analysis showed that preoperative RVD could provide reasonable classification of patients with prolonged CPB time and PLOS. It has been proved that prolonged CPB time and PLOS could reflect the adverse outcome in cardiac surgical patients (32–34). To some extent, microcirculatory impairment might play an important role in the outcome of cardiac surgery. Firstly, microcirculation is essential to provide oxygen and nutrients to tissues and is the main site of immunological response, regulation of homeostasis, and vasomotor tone control (35). Secondly, it has been stated that microcirculatory impairment was an independent predictor of morbidity and mortality in several acute pathological conditions, such as cardiac surgery and septic shock (36, 37). However, the association between retinal microcirculation and clinical outcome of CHD patients undergoing cardiac surgery remains unknown. Our results might provide valuable insights into the predictive value of retinal microcirculation in the outcome of congenital cardiac surgery.

As previously documented, impairment of retinal microvasculature was associated with cardiac structure and function (38). Hence, we speculated the association between impaired retinal microcirculation and adverse surgical outcome might reflect sicker patients with lower cardiac output. However, our results indicated that microcirculatory variables were not associated with macrohemodynamic parameters in CHD patients undergoing cardiac surgery. Also, previous studies found that microcirculatory impairment could be observed even when systemic hemodynamics were within satisfactory limits in patients undergoing cardiac surgery (27, 39). This could be explained by the different anatomical structures and physiological regulation mechanisms of macro- and micro-circulation. On one hand, macro- and micro-vessels respond differently to therapeutic interventions and inflammatory molecules (40). On the other hand, cellular sensing mechanisms needed to maintain the microvascular autoregulation were damaged in patients with cardiac surgery (41). Notably, microvascular dysfunction might play an important role in the development of postoperative organ dysfunction but the change in microvascular perfusion was independent of the change in systemic hemodynamics. Therefore, it is essential to monitor the microcirculation of patients with cardiac surgery.

In the current context, more and more clinical trials have sought to evaluate the microcirculatory changes in patients undergoing cardiac surgery. Technological advancements made it possible to have real-time imaging of microcirculation. Several methods have been used to visualize microcirculation, such as video microscopy, near-infrared spectroscopy, and laser Doppler flowmetry. OCTA was a newly developed, reproducible and reliable microcirculation monitoring tool as shown by recent studies (42, 43) and the time required for a single blood flow imaging scan of OCTA is around 2 to 3 seconds, providing a non-invasive and rapid tool for assessment of the microcirculation in patients undergoing cardiac surgery. As previously reported, assessment of RVD using OCTA could offer a window to evaluate the systemic microvasculature (44, 45). For this reason, the implementation of OCTA might provide potential information into the outcome of patients undergoing cardiac surgery, assisting physicians to frame more customized and precise therapeutic decisions for the patients. Studies comparing the current methods and OCTA in the quantification of microcirculation are needed to investigate the value of adopting OCTA as a clinical tool for the CHD patients.

The main strength of our prospective study is the use of a state-of-the-art technique, namely OCTA, to observe the changes of retinal microcirculation in CHD patients undergoing cardiac surgery with CPB. Nonetheless, we acknowledge several limitations in our study. Firstly, we did not evaluate RVD during and immediately after surgery due to technical issues. In the future, a portable handheld OCTA instrument could be used to explore the application of OCTA during and soon after surgery (46, 47). Secondly, a relatively small sample of CCHD patients was studied, which might have limited power for non-significant associations. Thirdly, the rate of the outcome events like mortality, neurological injury and end-organ damage was relatively low in the present study, limiting the power of our statistical analyses to assess the association between retinal microcirculation and these outcome events. In addition, we did not compare the differences in RVD changes for patients with different types of cardiac lesions and different severity due to the small sample size. Also, the study population was mostly elderly pediatric congenital heart disease patients, which could not represent for the whole perspective of congenital heart disease. Future studies with larger and multi-center sample sizes are needed.

## Conclusions

Our study results showed that RVD was improved after congenital cardiac surgery and impaired preoperative retinal microcirculation was associated with prolonged CPB time and PLOS, indicating OCTA could be used to identify potential adverse outcome due to prolonged CPB or PLOS and providing a novel non-invasive and rapid microcirculation monitoring tool for patients undergoing cardiac surgery.

## Data Availability Statement

The original contributions presented in the study are included in the article/Supplementary Material, further inquiries can be directed to the corresponding author/s.

## Ethics Statement

The studies involving human participants were reviewed and approved by the Research Ethics Committee of Guangdong Provincial People's Hospital [No.GDREC2018148H(R1)]. The patients/participants (or their parent or legal guardian in the case of children under 16 years) provided their written informed consent to participate in this study.

## Author Contributions

XY, HYu, CL, ZZ, and HYua: conception, design, drafting the article or revising it, and providing intellectual content of critical importance to the work described. CL, ZZ, HYua, PZ, QP, XD, MH, BL, YR, YK, XZ, HYu, and XY: analysis and interpretation of data. All authors: final approval of the version to be published.

## Conflict of Interest

The authors declare that the research was conducted in the absence of any commercial or financial relationships that could be construed as a potential conflict of interest.

## Publisher's Note

All claims expressed in this article are solely those of the authors and do not necessarily represent those of their affiliated organizations, or those of the publisher, the editors and the reviewers. Any product that may be evaluated in this article, or claim that may be made by its manufacturer, is not guaranteed or endorsed by the publisher.
