# Supplementary material for: Improved Retinal Microcirculation After Cardiac Surgery in Patients With Congenital Heart Disease
Source: Front Cardiovasc Med. 2021 Aug 31;8:712308. doi: 10.3389/fcvm.2021.712308 (PMC8438171; doi:10.3389/fcvm.2021.712308)
Supplement: Supplementary file 1 [file Image_1.PDF]

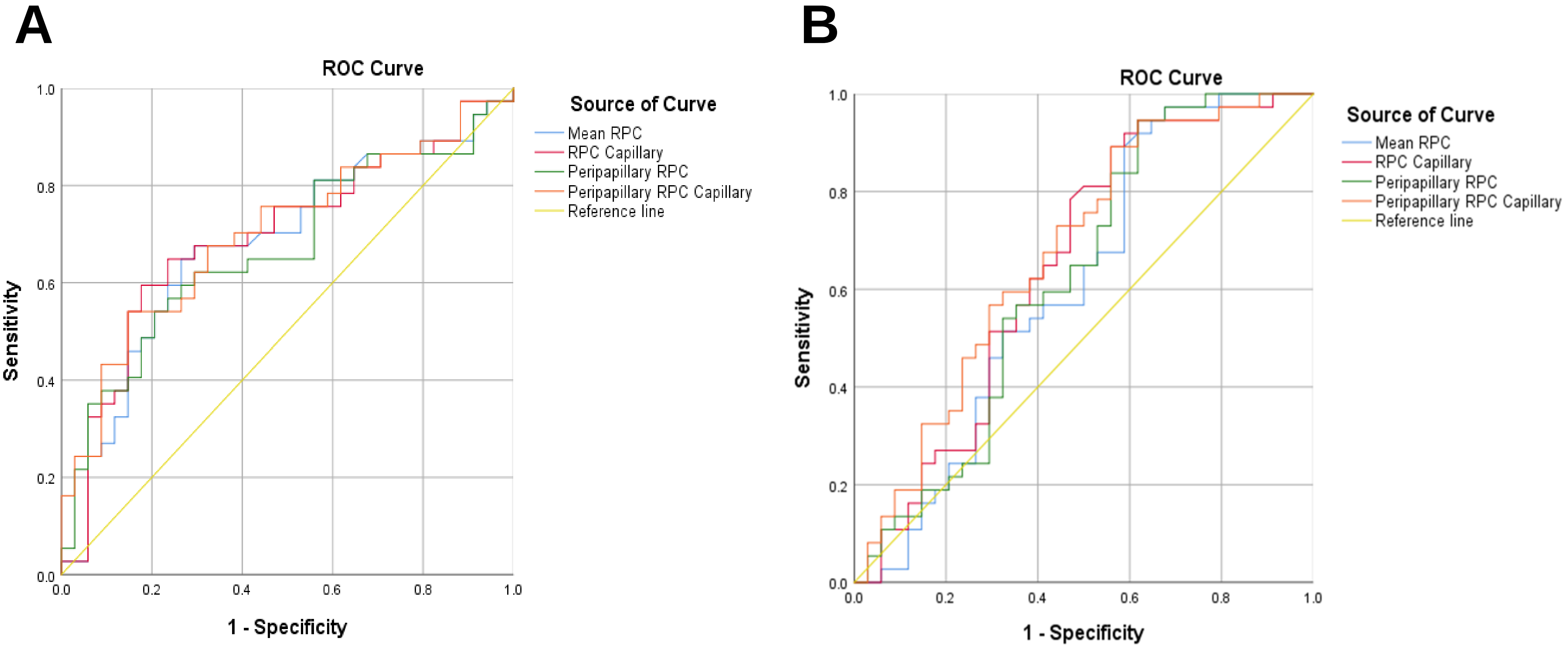
A
ROC Curve
Sensitivity
1 - Specificity
0.0
0.2
0.4
0.6
0.8
1.0
Source of Curve
Mean RPC
RPC Capillary
Peripapillary RPC
Peripapillary RPC Capillary
Reference line
B
ROC Curve
Sensitivity
1 - Specificity
Source of Curve
Mean RPC
RPC Capillary
Peripapillary RPC
Peripapillary RPC Capillary
Reference line

Figure legends

Supplementary Figure 1. ROC analysis of RVD in identifying patients with prolonged CPB time (A) and PLOS (B). The AUC of the VD of mean RPC, RPC capillary, peripapillary RPC and peripapillary RPC capillary to identify patients with prolonged CPB time is 0.679, 0.695, 0.670, and 0.702, respectively (A). The AUC of the VD of mean RPC, RPC capillary, peripapillary RPC and peripapillary RPC capillary to identify patients with prolonged PLOS is 0.611, 0.650, 0.626, and 0.676, respectively (B).

Abbreviations: ROC, receiver operating characteristic curve; RVD, retinal vessel density; CPB, cardiopulmonary bypass; PLOS, postoperative length of stay; AUC, area under the receiver operating characteristic curve; RPC, radial peripapillary capillary.
